# Knowledge, Attitude, Practices, and Sources of Information (KAPS) Toward COVID-19 During the Second Wave Pandemic Among University Population in Qatar: A Cross-Sectional Study

**DOI:** 10.3389/fpubh.2022.906159

**Published:** 2022-06-20

**Authors:** Ibrahim Alkaabi, Magdy Abita, Yousif Mahdi, Amr Ouda, Mohammed Imad Malki

**Affiliations:** ^1^College of Arts and Sciences, Deanship of General Studies, Qatar University, Doha, Qatar; ^2^Social Work Program, Department of Social Science, College of Arts and Sciences, Qatar University, Doha, Qatar; ^3^Psycology Program, Department of Social Science, College of Arts and Sciences, Qatar University, Doha, Qatar; ^4^College of Medicine, QU Health, Qatar University, Doha, Qatar

**Keywords:** COVID-19, knowledge, attitude, practice, sources, Qatar

## Abstract

**Background:**

The World Health Organization (WHO) declared COVID-19 as a pandemic on 11 March 2020. Many efforts were performed to contain the virus worldwide. People's knowledge and attitude should be directed toward strict preventive practices to halt the spread of the virus. We aimed to assess the knowledge, attitude, practices, and sources of information (KAPS) used by Qatar University (QU) attendees.

**Methods:**

A cross-sectional web-based questionnaire was answered by 500 employees and students in the QU community. It included questions on KAPS toward COVID-19. Information on sociodemographics was collected and analyzed. This study was conducted during the second wave of COVID-18 in the state of Qatar (April–May 2021).

**Results:**

A total of 475 participants aged between 18 and 68 years old consented to complete the survey questionnaire. The study involved 279 (58.7%) non-Qatari nationals and 196 (41.3%) natives, with 254 (53.5%) participants pursuing postgraduate studies and 221 (46.5%) undergraduates. Approximately two-thirds of the sample were employed (64.8%), while one-third were unemployed (35.2%). Knowledge scores on average were 66.4% (*M* = 5.31, *SD* = 1.45, and range: 0–8), with only significant differences were noted between nationalities (natives and non-natives) Participants' average score in practices was 69.72% (*M* = 4.18, *SD* = 1.7, and range 0–6) with a significant difference in safe COVID-19 practice scores based on the educational level. Adherence with COVID-19 policies and rules were 82% (*M* = 2.46, *SD* = 0.7, and range: 0–3) with no differences noted between groups. In addition, the population reported relying on governmental press conferences (76.0%) as their primary source of gaining details concerning COVID-19, followed by social media (64.4%). The least popular resources were information gained from family, relatives, friends, and coworkers (47.4%) and the news channels on TV (46.7%).

**Conclusion:**

Overall, this study provides insights into Qatar's KAPS toward COVID-19 during the quarantine of the second wave of this pandemic. This study, being the first of its kind to be conducted in the state of Qatar, is expected to help the ministry of public health and the government communication office to establish a suitable measurement of response to the spread of COVID-19 and develop the best practices for any future epidemics that might occur.

## Introduction

The coronavirus (CoV) is a large single-stranded RNA virus known to cause many human and animal diseases (1). Many well-known disease epidemics, especially in the Middle East region, have been attributed to CoV (2). These include Middle East respiratory syndrome coronavirus (MERS-CoV) and severe acute respiratory syndrome coronavirus (SARS-CoV) (2). In December 2019, multiple cases of pneumonia were recognized in Wuhan, China (3). The pathogen responsible for causing these clinical conditions was later identified as a novel member of the coronavirus family that was named severe acute respiratory syndrome coronavirus 2 (SARS-CoV-2) (3). As the disease has since spread rapidly in China and globally, the World Health Organization (WHO) designated the disease as a pandemic in March 2020 (4). By the end of 2020, coronavirus disease 2019 (COVID-19) has infected ~85 million people and led to more than 1.8 million deaths (5). The disease causes various symptoms, ranging from mild, unnoticeable symptoms to hospitalizing critical symptoms (6). Furthermore, a systematic review of population-based studies and cross-sectional surveys estimated that one-third of patients with COVID-19 are asymptomatic (7). This huge number of asymptomatic patients poses an additional challenge in controlling the spread of the disease, as it is believed that asymptomatic patients can transmit the disease as well (8).

Various safety measures were implemented internationally to fight against COVID-19. The goal of these measures was mainly to decrease the transmission of the disease and avoid overloading the healthcare system of countries (9). The severity of these actions ranged widely from non-mandatory social distancing recommendations to complete lockdowns with variable degrees of success (10). The state of Qatar has implemented multiple policies as soon as the first COVID-19 case was discovered in late February 2020 (11). These measures included travel restrictions, closure of restaurants, cafes, and public areas, and suspension of face-to-face education in schools and universities (11). Qatar then invested a lot in raising public awareness through educating the community about public preventive measures (12). These awareness campaigns were heavily spread through physical advertising banners and social media as well (13).

However, a clear understanding of the level of public awareness after such campaigns is crucial for policymakers in their fight against the upcoming waves of the COVID-19 pandemic (14). This study evaluates the awareness of the multi-cultural population residing in Qatar through a survey assessing the knowledge, attitude, practice, and sources of information (KAPS) toward COVID-19. Our study aims to assess the KAPS of the Qatari population toward COVID-19 and explore its relation to many factors, such as the demographics of the population. Unlike the commonly accepted KAP studies that have been published recently in different countries, the research team decided to include a “source of information” section in the survey. The aim of this was to assess where the public's awareness of the pandemic stems from in the country.

## Materials and Methods

A cross-sectional web-based questionnaire was developed to support the social work and psychology programs that are part of the department of social sciences and medical specialists in the College of Medicine to measure the KAPS.

The questionnaire made was distributed through the university's official email portal to faculty, students, and staff between April and May 2021. It was compromised of true/false, yes/no/sometimes, and checkbox types of questions. Every participant had the right to withdraw from the study even after completing it. Qatar University (QU)'s strict participant confidentiality policy was upheld throughout this research.

### Participants

All included participants were older than 18 years and had to be enrolled in the QU either as students, staff, or faculty. The English language was necessary to answer the survey. Finally, a decision to only include residents in Qatar since at least February 2020 was taken by the research team to avoid information bias by new residents of the country.

The inclusion criteria were as follows:

- being older than 18 years of age
- currently a student, staff, or faculty at the QU
- capable of reading English
- has been in Qatar since at least February 2020.

The study's exclusion criteria were only:

- Full consent is not provided by the subject
- The subject does not fully complete all the questions.

### The Survey

The developed survey was inspired by multiple similar COVID-19 questionnaires and input from behavioral scientists from the QU (as shown in Supplementary Material). The survey took approximately 8 min to be completed and was only sent in English. It was separated into five main components, each assessing a different aspect of the research. The first part was answered by checking a box that collected general demographic data about the participants, such as gender, age, nationality (Qatari or non-Qatari), an education level (undergraduate or postgraduate), and employment status. The second part, compromised of eight questions, assessed participants' knowledge through true and false statements regarding COVID-19. Statements started as general knowledge and got more specific each time.

Parts three, four, and five were all developed using the same yes/no/sometimes format, with each part targeting a different component of our research question. Part three focused on the practices of the public. Questions included the usage of hand sanitizer, washing hands with soap for 20 s, wearing facemasks in public, avoiding gatherings altogether, appropriate social distance, and attending social events or gatherings regardless of indoors or outdoors. Part four assessed the attitude of the population toward the COVID-19 laws introduced by the government of Qatar, which included questions, such as whether the national case tracking app (Ehteraz) was used, whether the participants reported suspicious symptoms of themselves, colleagues, friends, and family members to the government. Furthermore, whether QU's internal laws regarding COVID-19 were followed. Finally, and unlike a lot of other surveys on the matter of assessing the KAP only, the research team decided to include the sources of information most used by the public. The question asked participants to indicate with a yes/no or sometimes whether they use the following outlets as sources of information. The outlets included were news channels, direct governmental press conferences, social media or family, friends, relatives, and coworkers.

After the investigators developed the questionnaire, face-validity was confirmed by four public experts at the QU. They assessed and validated the instrument, providing several suggested modifications to improve the content and clarity of the questionnaire. Then, to measure the internal consistency (reliability coefficient) of the questionnaire and to detect any flaws with the survey, the link for the questionnaire was piloted on nine subjects. A Cronbach's alpha score of α = 0.715 was found, which is a satisfactory internal validity score according to Bland and Altman (15).

### Statistical Analysis

An independent sample *t*-test, one-way analysis of variance (ANOVA) test of significance, and multiple linear regression were used to examine the relation between the demographic characteristics and variables. A Pearson correlation analysis was used to compare correlations between variables. All statistical analyses were performed using IBM SPSS 28.

## Results

### Demographics

Out of 491 participants who took part in this investigation, 475 participants consented to complete the survey questionnaire. Among the final sample, 308 (64.8%) were women, and 167 (35.2%) were men, aged between 18 and 68 years old. The study involved 279 (58.7%) non-Qatari nationals, and 196 (41.3%) natives. Further, 254 (53.5%) participants pursued postgraduate studies, and 221 (46.5%) were undergraduates. Approximately two-thirds of the sample were employed (64.8%), while one-third were unemployed (35.2%). Demographic characteristics are shown in Table 1.

**Table 1:** Demographic characteristics of the subjects included in this investigation.

**Characteristics**		**Numbers of participants (%)**	
		***N***	***%***
Gender	Male	167	35.2%
	Female	308	64.8%
Age	18–29	215	45.3%
	30–49	188	39.6%
	50+	72	15.2%
Nationality	Qatari	196	41.3%
	Non-Qatari	279	58.7%
Education	Undergraduate	221	46.5%
	Postgraduate	254	53.5%
Occupation	Employed	308	64.8%
	Unemployed	167	35.2%
Total	–	475	100%

### COVID-19 Knowledge

Regarding the participant's background knowledge of COVID-19 based on true or false questions, the subjects demonstrated a proficient level of understanding. The subjects scored on average 66.4% (*M* = 5.31, *SD* = 1.45, and range: 0–8). Based on independent sample *T*-tests (for gender, nationality, education, and occupation), and an ANOVA (for age), there was a significant difference in scores for nationality (*p* ≤ 0.01), with findings suggesting that non-Qatari residents scored higher (*M* = 5.61, *SD* = 1.0) than Qatari nationals (*M* = 5.31, *SD* = 1.2) (as shown in Table 2). The correct answer rate across all the eight COVID-19 knowledge questions ranged between 18.3 and 96.2%. The most accurate and correctly answered question was “People who are older or have certain underlying medical conditions are at higher risk of getting more seriously ill from COVID-19” (97.9%). The least correctly answered question was “Supportive care is the current treatment for COVID-19” (15.4%) (as shown in Table 2).

**Table 2:** Coronavirus disease 2019 knowledge scores by demographic variables.

**Characteristics**		**COVID-19 Knowledge Scores (mean ±standard deviation)**	***p*-value**
Gender	Male	5.56, 0.9	*p* **>** 0.05
	Female	5.44, 1.1	
Age	18–29	5.52, 1.1	*p* > 0.05
	30–49	5.38, 1.1	
	50+	5.67, 0.9	
Nationality	Qatari	5.31, 1.2	*p* ≤ 0.01
	Non-Qatari	5.61, 1.0	
Education	Undergraduate	5.41, 1.2	*p* > 0.05
	Postgraduate	5.56, 1.0	
Occupation	Employed	5.49, 1.1	*p* > 0.05
	Unemployed	5.49, 1.1	
Total	–	5.31, 1.45	–

### Practice

Regarding the practice of COVID-19, most of the subjects indicated that they follow guidelines in keeping with safe COVID-19 practices across all six questions. As shown in Table 3, the participant's average score confirming full compliance with recommended COVID-19 practices was 69.72% (*M* = 4.18, *SD* = 1.7, and range 0–6). When stratifying the sample based on demographic characteristics, there was a significant difference in safe COVID-19 practice scores based on educational level (*p* ≤ 0.05); with findings suggesting that undergraduates score higher (*M* = 4.38 and *SD* = 1.6) than postgraduates (*M* = 4.05 and *SD* = 1.7). Responses kept in line with safe COVID-19 practices ranged between 67.5 and 73%. The most practiced COVID-19 factor was wearing a facemask in public places (90.5%). The least practiced indicator was attending large social gatherings indoors and outdoors (59.2%). Finally, the results revealed that participants mostly replied ‘Sometimes' toward social distance by at least 1.5 m (27.5%) (as shown in Table 3).

**Table 3:** Coronavirus disease 2019 safe practice scores by demographic variables.

**Characteristics**		**COVID-19 practice scores (mean ±standard deviation)**	***p*-value**
Gender	Male	4.16, 1.8	*p* > 0.05
	Female	4.22, 1.7	
Age	18–29	4.16, 1.7	*p* > 0.05
	30–49	4.26, 1.7	
	50+	4.19, 1.8	
Nationality	Qatari	4.26, 1.6	*p* > 0.05
	Non-Qatari	4.16, 1.0	
Education	Undergraduate	4.38, 1.6	*p* ≤ 0.05
	Postgraduate	4.05, 1.7	
Occupation	Employed	4.28, 1.7	*p* > 0.05
	Unemployed	4.06, 1.8	
Total	–	4.18, 1.7	–

### Attitude

As illustrated in Table 4, the results showed a favorable attitude toward the laws regarding COVID-19 conduct in Qatar and within QU. Findings suggest that subjects' average score confirming full adherence with COVID-19 policies and rules was 82% (*M* = 2.46, *SD* = 0.7, and range: 0–3). When statistically compared using the *T*-test (for gender, nationality, education, and occupation) and ANOVA (for age), there was no significant difference in the attitude score. All COVID-19 laws in the questionnaire were abided similarly among all groups. Across the different groups included in this study, responses in full adherence to COVID-19 laws (“*Yes*,” response) ranged between 80.3 and 83.7%. The most practiced law by the participants was using the Ehteraz track and trace application (95.6%), while the least practiced law was reporting symptoms of COVID-19 for themselves, family members, friends, or colleagues (26.5%). More details are shown in Table 4.

**Table 4:** Attitude toward COVID-19 laws and rules in Qatar by demographic variables.

**Characteristics**		**COVID-19 attitude toward laws scores (mean ±standard deviation)**	***p*-value**
Gender	Male	2.46, 0.8	*p* > 0.05
	Female	2.45, 0.7	
Age	18–29	2.47, 0.8	*p* > 0.05
	30–49	2.44, 0.8	
	50+	2.47, 0.8	
Nationality	Qatari	2.50, 0.7	*p* > 0.05
	Non-Qatari	2.43, 0.8	
Education	Undergraduate	2.51, 0.7	*p* > 0.05
	Postgraduate	2.41, 0.8	
Occupation	Employed	2.47, 0.8	*p* > 0.05
	Unemployed	2.43, 0.8	
Total	–	2.46, 0.7	–

### Source of Information

With regards to the most adopted means of getting information for COVID-19, data showed that the population in Qatar uses government press conferences (76.0%) as their primary source of gaining details concerning COVID-19, followed by social media (64.4%). The least popular resources were information gained from family, relatives, friends, and coworkers (47.4%) and the news channels on TV (46.7%) (as shown in Supplementary Material and Table 2).

### Regression Analysis

Findings from the multiple linear regression analysis models of the key indicators of this investigation (gender, age, nationality, education level, and occupational status) are demonstrated in Table 5. The results showed that knowledge scores as predicted by nationality were significantly affected [*F*_(5, 469)_ = 2.343, *p* < 0.05, and *R*^2^ = 0.014]. This suggests that nationality explains and predicts a significant amount of the variance in knowledge toward COVID-19, with non-Qatari residents scoring higher than their Qatari counterparts (β: 0.308, and *p* < 0.01). Multiple regression was also carried out to investigate whether the demographic factors significantly predicted COVID-19 safety practice scores. The results of the regression indicated that the model was significant, as predicted by the education level and occupation status [*F*_(5, 469)_ = 3.769, *p* < 0.01, and *R*^2^ =0.028]. Regarding educational level, postgraduates scored lower than their undergraduate counterparts (β: −0.896 and *p* < 0.01). In addition, unemployed subjects scored lower than those who were employed (β: −0.717 and *p* < 0.01). Finally, a multiple linear regression was calculated for total attitude toward COVID-19 law scores as predicted by key participant features, which showed that the model was not significant [F _(5, 469)_ = 1.462, *p* > 0.05, and *R*^2^ =0.005].

**Table 5:** Results of significant multiple linear regression on factors associated with COVID-19 knowledge and practices.

**Variable**	**Coefficient (β)**	**Standard error**	**F**	***p***	**95.0% Confidence Interval**	
					**Lower bound**	**Upper bound**
**Knowledge**			2.343	*p* <0.05		
Nationality (non-Qatari)	0.308	0.115			0.082	0.535
**Practice**			3.769	*p* <0.01		
Educational level (Postgraduate)	−0.896	0.230			−1.347	−0.445
Occupational status (Unemployed)	−0.717	0.238			−1.185	−0.249

## Discussion

This study is the first in Qatar during the COVID-19 pandemic that examines the KAPS among the general population. Similar articles examining KAP can be found in many countries, such as Syria, Bosnia and Herzegovina, and China (16–18). The research team decided to include the sources of information as well, considering its importance in the decision-making process.

Interestingly, the population of Qatar demonstrated a proficient level of understanding regardless of all factors (gender, age, education, nationality, and employment status), with a mean knowledge score of 5.31. Regardless, the only significant difference in knowledge scores was in nationality, as non-Qataris demonstrated a higher mean knowledge score than their Qatari counterpart. A possible explanation for this result could be that non-Qataris likely experienced previous pandemics, such as H1N1, SARS, or MERS in their native countries before. Qataris, on the other hand, have not been exposed much to such pandemics before as the country was never globalized like today ever before.

The knowledge score metric was overall very comparable among groups and relatively high with a mean score of 5.31 out of 8, which proves the success of Qatar's strategy of relaying information to the public as it was accessible to all people regardless of their age and education level, occupation status, and gender. Attitude toward the law across the population was similar regardless of gender, age, nationality, education, and occupation status. With a mean score of 2.46 out of 3, the population managed to impress with its adherence to laws. It is important to note that the severe penalties enacted by the government against COVID-19 lawbreakers might have introduced some bias to this study. While all participants were assured of complete anonymity, we cannot discredit the possibility of bias affecting the results, especially in the attitude toward laws section. This might explain why more than 92% of participants answered that they follow instructions issued by QU while only 90.5% reported wearing a face mask in public which is considered the main instruction provided by QU health. Regarding the practice toward COVID-19, most of the subjects indicated that they follow guidelines in keeping with safe COVID-19 practices, across all six questions. With a mean score of 2.46 out of 3, the population demonstrated a very mature approach toward COVID-19, with the only significant difference being among the education group, whereas postgraduates were less likely to follow the rules compared with undergraduates. It must be noted that Qatar's approach to face masks was probably why it was the most practiced indicator. Qatar's strategy, which included non-tolerable fines, constant surveillance, and continuous availability of PPE, seems to have proved successful.

In fact, compared with the data reported in many countries, such as Syria, Thailand, and China, the population in Qatar has shown a considerably high knowledge score compared with the population in these counterparts (16, 17, 19). For example, in Thailand, 73.4% had poor knowledge of COVID-19 (19). This underlines the importance of the Qatar national health authorities providing relentlessly clear updates and information about the emerging virus and the need to continuously assess and monitor whether their messages are being understood within the community. Furthermore, the vast majority of the population in Qatar is in agreement that all the preventive measures taken by the government is effective against COVID-19.

Our hypothesis was that social media would be the main source of information used by the public. Surprisingly, however, approximately three-quarters of the survey fillers noted that they get information directly from governmental press conferences. This result, when compared with the rest of the world, is unique (20, 21). The Qatari government has proved to be a leader in information campaigns regarding this pandemic. Therefore, their strategy deserves a closer and deeper look, perhaps in another article.

Over the past 2 years, many countries in the world have conducted the COVID-19 KAP study, as the results provide invaluable insight into prevention control and population management. In 2004, a similar article examined the KAP of the population in relation to SARS. In line with our article, the 2004 study also found that Qataris scored significantly lower than non-Qataris. In this regard, Qataris scored 31.7% in knowledge, while non-Qataris scored 68.3% (22). The explanation cited was that the Arabic-speaking community lacks information about SARS due to the vast majority of news coverage in English (22). This article is interesting to note because Dr Abdelatif Al Khal, the second author, is the Executive Director of Hamad Medical Corporation's Division of Infectious Diseases and Chair of the National Health Strategic Group on COVID-19 and all his governmental conferences are being conducted live, interactively, and in Arabic, a strategy that might have helped the Qatari governmental conferences to displace both social media and traditional news outlets as the main sources of information in the pandemic.

Another KAP article was published in Qatar during the COVID-19 pandemic. It measured the KAP of paramedics toward Personal Perspective Equipment than the disease itself (23). As the only study in Qatar that addressed the general population and used the KAPS tool, our article is the first of its kind in Qatar during the COVID-19 pandemic.

It is important to note that our study does not represent the university's population over the whole country. While QU is the biggest and the first national university in Qatar, other universities do exist in the country, and they were not added to the study. Therefore, the research team recommends that wider scale studies are conducted in the country to truly assess the population's KAPS and reduce the survey bias by increasing the number of participants.

## Conclusion

Overall, the population of the state of Qatar has demonstrated favorable results compared with the rest of the world and the region, especially regarding mask-wearing. These results explain how the country achieved an astonishing 0.18% death ratio from COVID-19. Only 8.14% of the total population ever contracted the virus regardless of over five million tests being made by January 2022 in the country of less than three million people (24–26). Qatar's response to the COVID-19 pandemic was excellent and deserved a closer look in a follow-up article. The results of this study might be generalizable to other regional countries in the Gulf Cooperation Council (GCC) that have experienced similar second wave of COVID-19 pandemics. This study presents a unique reference for pandemic cautious behavioral response to COVID-19 in a well-developed health system.

## Data Availability Statement

The original contributions presented in the study are included in the article/Supplementary Material, further inquiries can be directed to the corresponding author/s.

## Ethics Statement

The studies involving human participants were reviewed and approved by Qatar University Institutional Review Board (QU-IRB) Number: 1727764-1. Written informed consent for participation was not required for this study in accordance with the national legislation and the institutional requirements.

## Author Contributions

MM conceived and supervised the study. IA, MA, and MM designed the questionnaire, obtained IRB approval, and distributed the survey. YM analyzed the data. AO and MM drafted the manuscript. All authors revised the final manuscript. All authors contributed to the article and approved the submitted version.

## Conflict of Interest

The authors declare that the research was conducted in the absence of any commercial or financial relationships that could be construed as a potential conflict of interest.

## Publisher's Note

All claims expressed in this article are solely those of the authors and do not necessarily represent those of their affiliated organizations, or those of the publisher, the editors and the reviewers. Any product that may be evaluated in this article, or claim that may be made by its manufacturer, is not guaranteed or endorsed by the publisher.
