# Supplementary material for: Knowledge, Attitude, Practices, and Sources of Information (KAPS) Toward COVID-19 During the Second Wave Pandemic Among University Population in Qatar: A Cross-Sectional Study
Source: Front Public Health. 2022 Jun 20;10:906159. doi: 10.3389/fpubh.2022.906159 (PMC9251538; doi:10.3389/fpubh.2022.906159)
Supplement: Supplementary file 1 [file Table_1.DOCX]

Supplementary Material

# Supplementary Tables

**1.1 Questionnaire used for Awareness of COVID-19 during the second wave pandemic within the state of Qatar**

1. Sex

M 🞏 F 🞏

3. Age:

18-28 🞏 29-39 🞏 40-50 🞏 51-60 🞏 61 and above 🞏

4. Nationality:

Qatari🞏 Non-Qatari🞏

5. Educational level:

Undergraduate🞏 postgraduate🞏

6. Occupational status: employed 🞏 unemployed 🞏

**Please indicate if the following statements are ‘True’ or ‘False’:**

| **The first component: Knowledge** | | **True** | **False** |
| --- | --- | --- | --- |
| 1 | The incubation period of COVID-19 is 2-14 days |  |  |
| 2 | The most common COVID-19 symptoms are fever, dry cough, and tiredness? |  |  |
| 3 | People who are older or have certain underlying medical conditions are at higher risk of getting more seriously ill from COVID-19 |  |  |
| 4 | Supportive care is the current treatment for COVID-19 |  |  |
| 5 | Having a healthy diet is essential in maintaining your immune system if you have COVID-19 |  |  |
| 6 | Smokers are not effected any differently by COVID-19 compared to non-smokers |  |  |
| 7 | Using supplements is beneficial in preventing COVID-19 |  |  |
| 8 | Exercising regularly or playing sports will help in reducing the impact of COVID-19 |  |  |

**Please indicate if you practice the following:**

| **The second component: Practice** | | **Yes** | **No** | **Sometimes** |
| --- | --- | --- | --- | --- |
| 1 | Have you attended large social gatherings indoors and outdoors? |  |  |  |
| 2 | Do you socially distance by at least 1.5 meters from another person? |  |  |  |
| 3 | Do you avoid crowded gatherings indoors and outdoors? |  |  |  |
| 4 | Do you wear a facemask when in public at all times? |  |  |  |
| 5 | Do you wash your hands with soap and water for at least 20 seconds? |  |  |  |
| 6 | Do you use hand sanitizer often? |  |  |  |

**Have you done the following with regards to COVID-19 laws within the State of Qatar:**

| **The fourth component: Attitude towards COVID-19 laws** | | **Yes** | **No** | **Sometimes** |
| --- | --- | --- | --- | --- |
| 1 | Do you use the Ehteraz application as set out by the State of Qatar? |  |  |  |
| 2 | Do you report suspicious symptoms of COVID-19 for yourself, family members, friends or colleagues? |  |  |  |
| 3 | Do you follow the instructions issued by QU regarding COVID-19 conduct? |  |  |  |

**Please indicate which media resources you use to gain information regarding COVID-19:**

| **The fifth component: Resources** | | **Yes** | **No** | **Sometimes** |
| --- | --- | --- | --- | --- |
| 1 | News channels |  |  |  |
| 2 | Government press conferences |  |  |  |
| 3 | Social Media |  |  |  |
| 4 | Family, relatives, friends and coworkers |  |  |  |

**1.2 Summary of Questions for Knowledge, Practice, Attitude, and Resources Used Towards COVID-19.**

| **Questions** | **True** | **False** | **Yes** | **No** | **Sometimes** |
| --- | --- | --- | --- | --- | --- |
| Knowledge |  |  |  |  |  |
| *K1*. The incubation period of COVID-19 is 2-14 days | 87.8% | 12.2% |  |  |  |
| *K2*. The most common COVID-19 symptoms are fever, dry cough, and tiredness? | 97.3% | 6.3% |  |  |  |
| *K3*. People who are older or have certain underlying medical conditions are at higher risk of getting more seriously ill from COVID-19 | 97.9% | 2.1% |  |  |  |
| *K4*. Supportive care is the current treatment for COVID-19 | 84.6% | 15.4% |  |  |  |
| *K5*. Having a healthy diet is essential in maintaining your immune system if you have COVID-19 | 84.6% | 15.4% |  |  |  |
| *K6.* Smokers are not affected any differently by COVID-19 compared to non-smokers | 14.9% | 85.1% |  |  |  |
| *K7*. Using supplements is beneficial in preventing COVID-19 | 52% | 48% |  |  |  |
| *K8*. Exercising or playing sports will help in reducing the impact of COVID-19 symptoms | 63.8% | 36.2% |  |  |  |
| Practice |  |  |  |  |  |
| *P1*. Have you attended large social gatherings indoors and outdoors? |  |  | 14.3% | 59.2% | 26.5% |
| *P2*. Do you socially distance by at least 1.5 meters from another person? |  |  | 64.8% | 6.7% | 28.4% |
| *P3*. Do you avoid crowded gatherings indoors and outdoors? |  |  | 76% | 7.4% | 16.6% |
| *P4*. Do you wear a facemask when in public at all times? |  |  | 90.5% | 3.2% | 6.3% |
| *P5*. Do you wash your hands with soap and water for at least 20 seconds? |  |  | 69.9% | 9.5% | 20.6% |
| *P6*. Do you use hand sanitizer often? |  |  | 72% | 10.9% | 17.1% |
| Attitude towards laws |  |  |  |  |  |
| *A1*. Do you use the Ehteraz application as set out by the State of Qatar? |  |  | 95.6% | 2.7% | 1.7% |
| *A2*. Do you report symptoms of COVID-19 for yourself, family members, friends, or colleagues? |  |  | 65.9% | 26.5% | 7.6% |
| *A3*. Do you follow the instructions issued by Qatar University regarding COVID-19 conduct? |  |  | 92.4% | 3.4% | 4.2% |
| Resources |  |  |  |  |  |
| *R1*. News channels |  |  | 46.7% | 29.9% | 23.4% |
| *R2*. Government press conferences |  |  | 76% | 8.8% | 15.2% |
| *R3*. Social Media |  |  | 64.4% | 15.8% | 19.8% |
| *R4*. Family, relatives, friends, and coworkers |  |  | 47.4% | 16.8% | 35.8% |
